# Supplementary figures and images for: Elevated pyramidal cell firing orchestrates arteriolar vasoconstriction through COX-2-derived prostaglandin E2 signaling
Source: eLife. 2025 Apr 23;13:RP102424. doi: 10.7554/eLife.102424 (PMC12017770; doi:10.7554/eLife.102424)

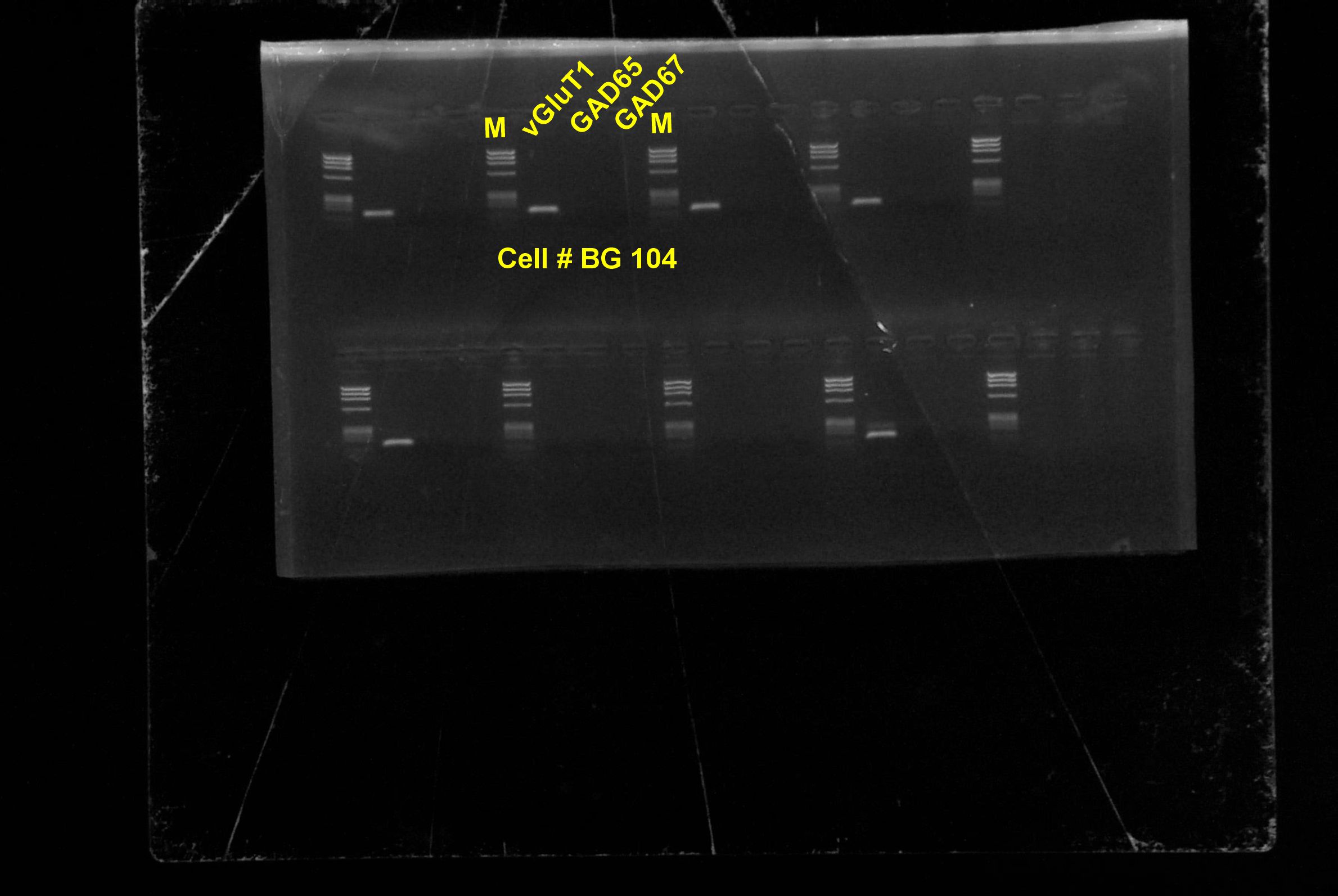

Supplement: Figure 4—source data 2. [file elife-102424-fig4-data2.zip › Figure 4 - source data 2a.jpg]

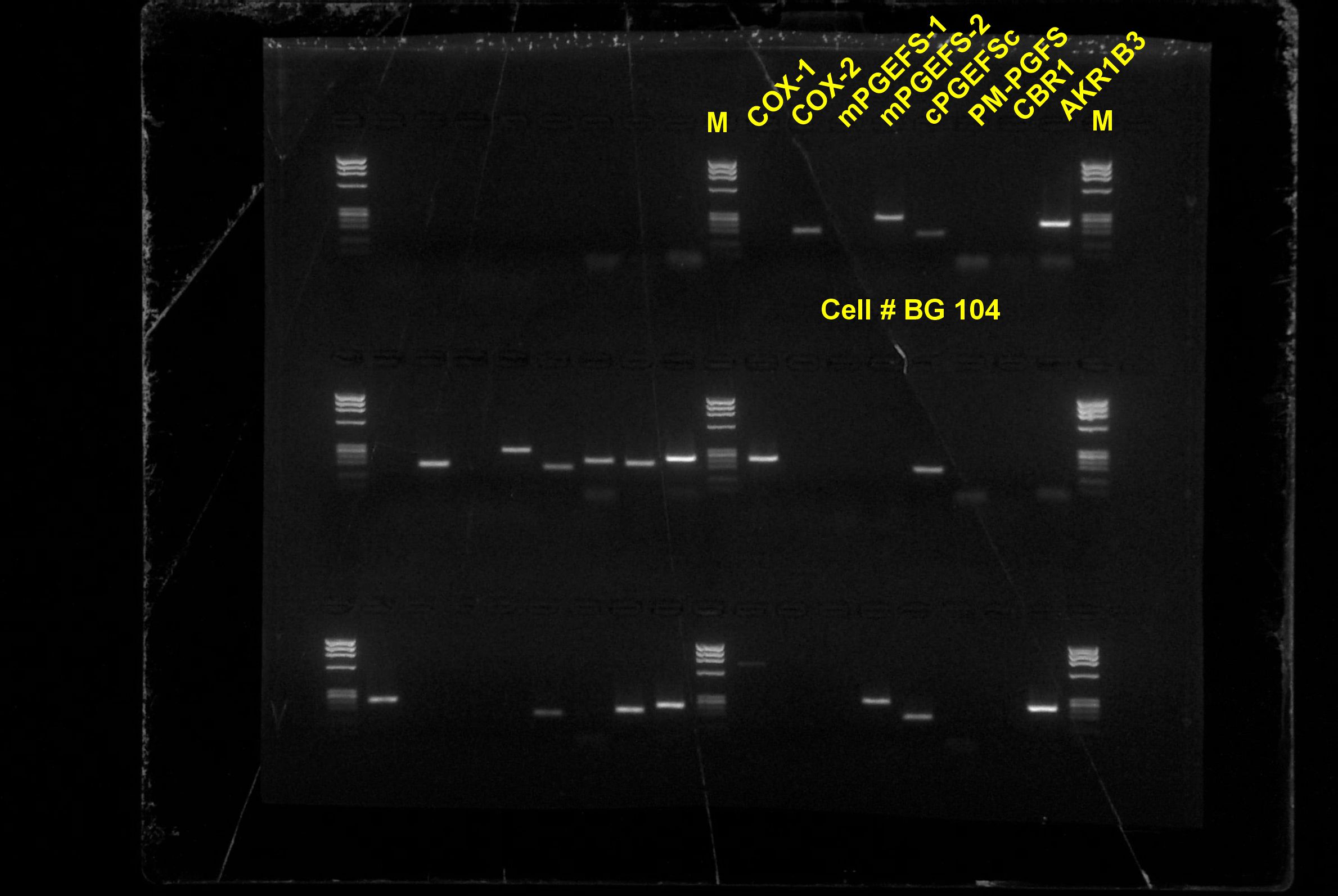

Supplement: Figure 4—source data 2. [file elife-102424-fig4-data2.zip › Figure 4 - source data 2b.jpg]

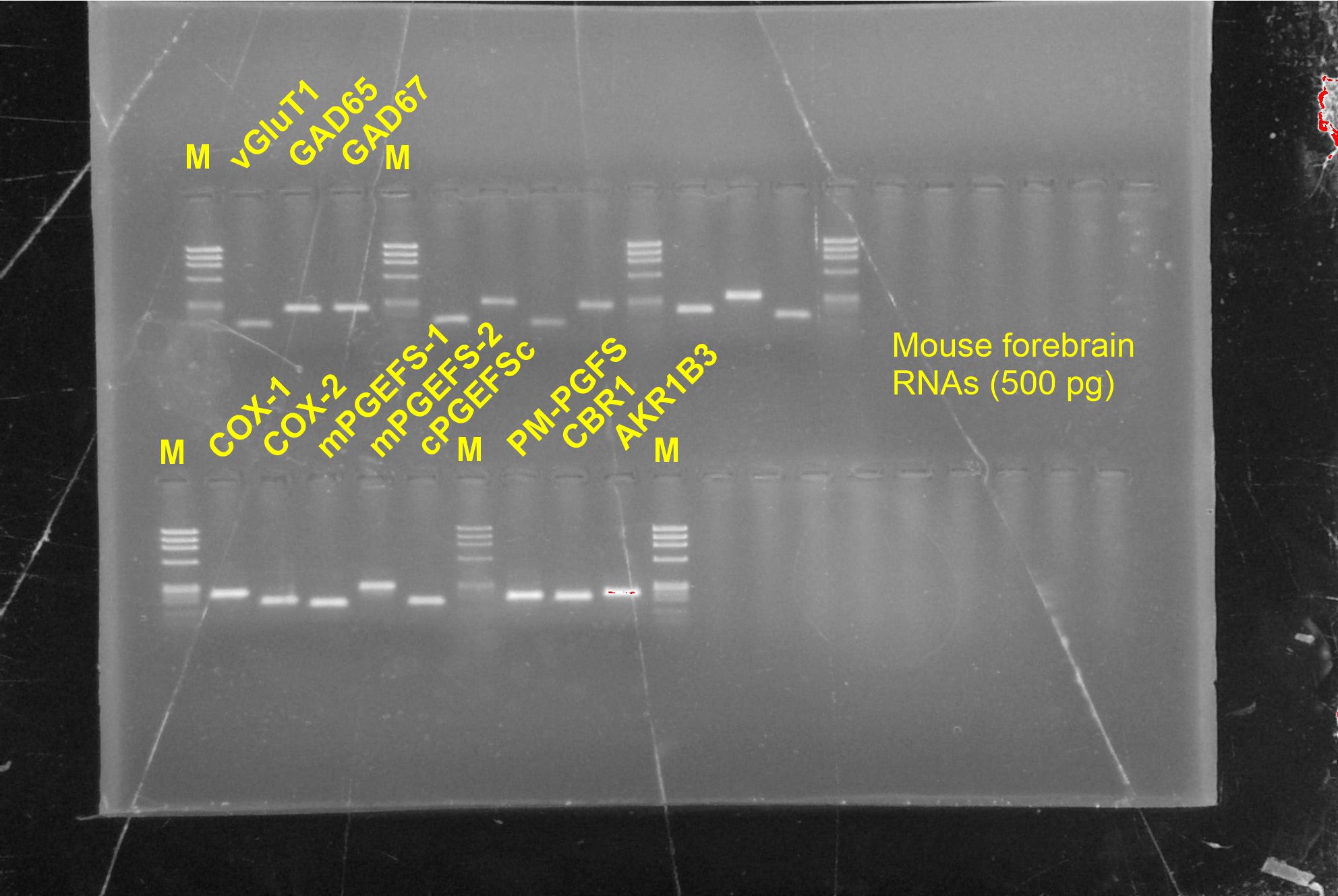

Supplement: Figure 4—figure supplement 1—source data 2. [file elife-102424-fig4-figsupp1-data2.zip › Figure 4 - figure supplement 1 - source data 2.jpg]
