# Supplementary material for: Elevated pyramidal cell firing orchestrates arteriolar vasoconstriction through COX-2-derived prostaglandin E2 signaling
Source: eLife. 2025 Apr 23;13:RP102424. doi: 10.7554/eLife.102424 (PMC12017770; doi:10.7554/eLife.102424)
Supplement: Source code 1. — The method has been described in Lacroix et al., 2015. [file elife-102424-code1.zip › Vessel Diameter Measurement Manual.pdf]

Automated Vessel Diameter Measurement

Getting Started

1. Running the software
    a. Start matlab and set the working directory to the folder that contains the vessel diameter tracking software (.fig and .m files).
    b. Enter ***vesselDiameter*** in the matlab command window.
2. Opening an image stack
    a. Press the “browse” button located in the top left corner of the window. A new search window will automatically open.
    b. Select the folder that contains the image stack.
    c. The contents of the folder will be displayed in the box beneath the browse button. Only files with the extension “.tif” or “.tiff” will be displayed as these are the only file types supported.
    d. Select the image file from the display box, this will load and display the first frame.
3. Rotating the image
    a. The detection algorithm assumes that the vessel is running vertically through the image therefore the image must be saved in the correct orientation or adjusted using the rotate button.
    b. Press the rotate button to rotate the image if necessary. This has limited functionality as it can only rotate 90 degrees.
4. Viewing the image stack
    a. The slidebar beneath the frame changes the displayed frame.
    b. Pressing the “Play” button located in the Movie panel plays the tiff series as a movie.
        i. The frame rate can be adjusted; however the rate is only an approximation. The movie algorithm works by loading the new frame and updating the display within a loop. Increasing the frame rate becomes limited by how fast Matlab can perform these actions.

Vessel Diameter Tracking

1. The vessel diameter is measured by tracking the position of the vessel walls through a series of images. Upon loading a stack of images, a line will be present in the center of the image. This line is used to select the region of interest (ROI).
2. Position the ROI line over the vessel such that the line crosses both walls of the vessel.

    ROI Line

    a. The ROI line can be moved by moving the endpoints or dragging the entire line.
        i. Move the endpoints by selecting (left mouse click and hold) an endpoint and dragging it to a new position.

        ii. Drag the entire line selecting non-endpoints of the line and dragging the entire line to a new positions
   b. When the ROI line is horizontal, the region of interest is composed of a single row of data.
   c. When the ROI line is not horizontal, the region of interest is an average of the data enclosed by a rectangle whose opposite corners are the endpoints of the ROI line. The averaging takes place down the columns with the resulting data have 1xn data points, where n is the number of columns in the ROI.
   d. Avoid making the ROI line vertical as this will cause an error due to too few data points.
3. Select the right and left vessel wall edges.
   a. The ROI data shows a plot of the data from the region of interest. In the images, the vessel walls are either bright (appearing as a peak in the ROI plot) or dark (appearing as a valley in the ROI plot).
   b. Use the left edge and right edge sliders to select the vessel wall edge.
        i. Note: The sliders are only enabled if the current frame is frame 1. This is to prevent inadvertent adjusting of the edge detection.
   c. Update the edge as being either a peak or a valley in the appropriate edge panel.
   d. You can turn on the distance line by pressing the "Distance Line Button". This places a white line in the image that represents the distance between the vessel walls. The endpoints of this line will move as you move the left and right edge sliders for the ROI data. Moving this line by clicking and dragging does *not* affect edge detection.
4. Track the vessel wall throughout the stack.
   a. Press the "Track Edges" button to start the automated vessel wall tracking.

Viewing the Results

1. While the tracking algorithm is running the plots will update showing the current image, the roi data (with detected peaks), and a plot of the vessel diameter in pixels.
2. When the tracking algorithm is finished ensure that the Distance is on Line (you can turn the ROI line off) and use the frame slider to view the results for an individual frame or play a movie that will update the distance line for each frame.

Manually Editing the Results

1. If the tracking results need to be manually adjusted, use the fame slider to select the frame in which the vessel wall is first incorrectly detected.
2. Press the edge "edit" button to enable the edge slider to allow manual editing of the edge position.
3. You can press the track edges button again and the edge tracking will start at the current frame and recalculate the positions of subsequent vessel walls.
   a. Be sure the update the edge valley/peak in the event that it has changed.
4. This process can be repeated.

Saving the Results

1. The vessel diameter distance that is plotted in the bottom plot can be saved.
2. Enter a file name in the save file name box.
    a. The file extension ".mat" will be added and should not be included in the file name box.
3. Press the "save" button.
    a. This will save the distance data in the same directory as the image file.
    b.
4. To subsequently load the data into the matlab workspace type "load fname" where fname is the name of the saved file. This will put a variable called guiOut in the workspace. guiOut is a structure with the fields filename and data. Filename is the name of the image stack file and data is the distance data.

    Example code to visualize:

    ```
    load fname;  % again fname should be changed to name of saved .mat file
    figure;
    plot(guiOut.data);
    xlabel('frame #');
    ylabel('diameter (inpixels)');
    ind = find(guiOut.filename == '\');
    ind = ind(end)+1;
    titlestr = guiOut.filename(ind:end);
    title(titlestr);
    ```
